# Primary Care Nurses’ Experiences of Structured Documentation: A Qualitative Interview Study

**DOI:** 10.1177/23333936251330684

**Published:** 2025-04-12

**Authors:** Anna Dalsten Hjort, Tora Hammar, Karin Myrberg

**Affiliations:** 1 Centre for Research and Development, Region Gävleborg/Uppsala University, Uppsala, Sweden; 2 Department of Medicine and Optometry, Faculty of Health and Life Sciences, eHealth Institute, Linnaeus University, Kalmar, Sweden; 3 Department of Caring Sciences, Faculty of Health and Occupational Studies, University of Gävle, Gävle, Sweden

**Keywords:** structured documentation, nursing, electronic health record, qualitative research, documentation quality, nursing workload, Sweden

## Abstract

Healthcare is undergoing an unprecedented technological transition to structured documentation in electronic health records (EHR), which has the potential to increase the quality of documentation. However, given the rising demand for direct transfer of data, there is a risk that requirements for more documentation will follow. This study seeks to investigate primary care nurses’ experiences of structured documentation with direct transfer to a national quality registry. Nine primary care nurses using structured documentation in their management of chronic obstructive pulmonary disease (COPD) patients were recruited from different Swedish regions. The semi-structured interviews addressed experiences and work procedures when using a structured documentation template with direct data transfer to a quality register. Interviews were transcribed verbatim and analyzed using qualitative content analysis. Data were framed according to five key concepts; patient safety, time-saving work methods, quality of care, equitable care, and professional autonomy. The nurses experienced some barriers in relation to structured documentation but mainly observed benefits, raising the potential to enhance equitable care and safety for patients with COPD in primary care. Professional experience and autonomy were described as important prerequisites in achieving these benefits. The findings from this study can contribute to strengthening the documentation work procedures of nurses.

Accurate healthcare documentation is critical in providing patients with quality care. Medical records serve as a vehicle for clinical communication between healthcare professionals, but are also a source of information for patients. Reuse of healthcare data for various purposes is an area of emerging interest. Additionally, medical records can be used for other purposes such as finance, legal issues, research and quality improvement (Häyrinen et al., 2008; Rosenbloom et al., 2011).

Increased use and regulatory requirements mean that nurses are spending more time on documentation and development of tools and techniques to increase documentation efficiency is ongoing (Ebbers et al., 2022). One example of this is structured and standardized documentation *(hereafter: structured documentation)* in Electronic Health Records (EHR) that enables reuse of data as well as automatic transfer to other systems. Structured documentation adheres to a highly organized format involving predefined semantic standards for templates as well as for data entries (Rosenbloom et al., 2011). The focus of this study is understanding registered primary care nurses’ experiences of such structured documentation in everyday clinical practice.

Incomplete and insufficient documentation affects patient management, continuity of care and medicolegal issues (Considine et al., 2016). Besides adverse patient outcomes, poor documentation may influence healthcare professionals’ decision-making abilities (Workie et al., 2023). Duplicated information, incorrect insertions and bloated notes, which are documents or notes containing large amounts of textual redundancy, are among the widely recognized problems that contribute to poor documentation (Ebbers et al., 2022). Structured documentation can serve as a means to overcome such problems, as it is associated with increased quality in documentation of patient processes due to the consensus it provides in relation to concepts and terms (Narayanan et al., 2017). It can also be reused for quality improvement purposes by direct transfer/migration to quality registries (Fredriksson et al., 2017). Unstructured free text data, with no particular structure or standard for how the information should be written and managed, is still the most common way to enter information (Rosenbloom et al., 2011). Free text information is considered well-suited for targeting patients’ complex difficulties and comorbidities (Vest et al., 2017), as well as for including patients’ own views on their health journey (Jefferies et al., 2010). Automatic transfer of free text data to other systems is possible to some extent, however, it is complicated as clinical texts vary with different individuals and contexts (Sun et al., 2018).

Healthcare documentation consequently constitutes a substantial proportion of nurses’ work, with administrative tasks taking time away from direct patient care. A complex and necessary shift to more modern information systems that facilitate structured healthcare documentation is taking place in Sweden and internationally. Due to this transformation, new working procedures are essential. Even though studies vary, it has been demonstrated that nurses spend about 40% of their time on healthcare documentation (De Groot et al., 2022, and that high administrative workloads increase the risk of stress and burnout (De Groot et al., 2022; Schenk et al., 2017). Previous studies have demonstrated that nurses perceive documentation in EHR to create high workload (Bøgeskov & Grimshaw-Aagaard, 2019; Fraczkowski et al., 2020). However, they were mainly positive about the task overall, as they perceived it to be an integral and meaningful part of care (Michel et al., 2017) De Groot et al. (2022) The challenges discussed include duplicated information (Bøgeskov & Grimshaw-Aagaard, 2019), mismatches between EHR terminologies and professional vocabulary (Lavin et al., 2015) and the difficulties involved in removing mandatory sections (De Groot et al., 2022). However, little is known about nurses’ views on structured documentation in relation to this issue, for example, regarding data transfer to national quality registries. The aim of the present study was thus to contribute to this emerging discussion by exploring primary care nurses’ experiences of structured healthcare documentation in their meetings with chronic obstructive pulmonary disease (COPD) patients.

## Method

The study employed an explorative qualitative approach (Sandelowski, 2010), with data collected through semi-structured interviews. A hermeneutic perspective (Gadamer, 2004) was adopted to interpret the underlying meanings and contexts within the data. The data was subsequently analyzed primarily through deductive and inductive qualitative content analysis, in accordance with the description by Elo and Kyngäs (2008). The hermeneutic circle is well suited for the purpose of understanding and interpreting the clinical experiences of asthma and COPD nurses. There is no absolute truth; rather, it is about reflecting the nurses’ perceived everyday life through structured documentation and the contexts they encounter in relation to other healthcare providers and patients. New insights can be identified through the analyzing the nurses’ narrative words. One of the authors possessed pre-understanding as an asthma and COPD nurse, which is an important aspect of hermeneutics, as it involves utilizing one’s own experience. This perspective was combined with the two other authors, who lacked this experience, and could be more neutral toward the written word (Gadamer, 2004).

This study is grounded in key concepts (patient safety, time-saving working methods, quality of care, equitable care) that are pivotal for good and safe care enabled, amoung other things, by efficient information management, as outlined by the Swedish Knowledge Governance Framework (Sveriges kommuner och regioner (SKR), NSG strukturerad vårdinformation, 2023.). Standardized and structured information is a prerequisite for safe and efficient information management, and together they can provide the foundation for opportunities for knowledge support and follow-ups on healthcare outcomes (Ebbers et al., 2022). Swedish Knowledge Governance Framework is a national system for health and medical care in Sweden. This system involves collaboration between Sweden’s local authorities and regions, the National Board of Health and Welfare, and the Swedish government, among others (Sveriges kommuner och regioner (SKR), NSG strukturerad vårdinformation, 2023.). The key concept, professional autonomy emerged from the public debate on the standardization of Swedish Knowledge Governance Framework (Cizinsky et al., 2019).

### Study Context

This study involves primary care nurses specialized in COPD patients. COPD is a disease that causes airflow blockage and breathing problems. In Sweden, stable patients with COPD are managed in primary care. The standard procedure is annual check-ups in which a specialist nurse performs spirometry, supports self-management and reviews the patient health status (Smith et al., 2023). Patient outcomes are reported to the Swedish National Airway Register (SNAR), a national quality register that includes both asthma and COPD patients. The registry, which has a coverage rate of 89%, aims to improve patients’ quality of care and quality of life. At present, 88% of all registrations in SNAR are directly transferred from the EHR system (Luftvägsregistret, 2021). Although the nurses enrolled in this study used different EHR systems for their structured documentation of COPD consultations, templates were quite similar, following the Swedish national guidelines for COPD management, which thus constitutes a rationale to involve this study group. Depending on the system, structured data entries were made using drop-down menus with selectable options or smart forms featuring interactive templates and checkboxes. Additionally, all systems allowed free text information to some extent.

### Recruitment and Participants

Inclusion criteria for participating primary care COPD nurses were: (a) that participants used an EHR system that enabled automatic data transfer to SNAR and (b) at least 1 year of experience of working with COPD patients in order to be familiar with the documentation procedure. Participants were contacted through snowball sampling, where the request was forwarded from the SNAR contact persons to their COPD networks and interviewees subsequently recommended other nurses from among their acquaintances to participate. Voluntary participants contacted the first author by e-mail and scheduled the interviews at a time convenient for the participant. Participants were recruited until a reasonable range of experiences had been captured in order to address the aim of the study. Nine nurses from nine different primary care units in four different Swedish health care regions participated in the study (participant information provided in Table 1). The goal was to recruit nurses using a range of EHR systems. Informed consent was obtained and participant confidentiality was stressed before the interviews started. All interviews were conducted via Microsoft Teams video meetings, and participants were able to choose whether to be at home or in the workplace. Time was available after the interviews for the participants to reflect and ask questions on what they had said.

**Table 1.** Overview of Study Participants.

Participant	Sex	Years of nursing experience (range 10–36)	Years of experience as chronic obstructive pulmonary disease (COPD) nurse (range 1–13)	Length of interview (min) (range 32–59)
1	F	36	6	59
2	F	24	6	39
3	F	36	12	40
4	F	20	1.5	32
5	M	15	8	57
6	F	14	7	53
7	F	12	3	46
8	F	10	1	40
9	F	25	13	55

### Data Collection

The interviews were guided by a semi-structured interview guide (see Appendix 1) developed by the research team. The open-ended questions were based on five key concepts frequently described as related to structured documentation; patient safety, time-saving working methods, quality of care, equitable care and professional autonomy (Cizinsky et al., 2019; Sveriges kommuner och regioner (SKR), NSG strukturerad vårdinformation, 2023; Törnvall & Wilhelmsson, 2008), and were formulated in order to obtain statements of relevance for the aim of the project. Examples of probing questions were “Please tell me more about your clinical documentation workflow” or “Can you tell me about how you experience structured documentation as compared to more general keywords and free text?” Follow-up questions were asked in order to obtain a more in-depth understanding of the participants’ statements. All interviews were conducted by the first author (ADH), a female with experience as a COPD nurse in primary care, who currently works in health informatics with a focus on EHR development. The second author (TH), also female, has extensive experience in research informatics. The third author (KM), another female, has expertise in healthcare implementation research and practical experience in EHR development. Data were collected over a period of 2 months in early 2023. The average interview time was 47 min (median 46). All interviews were digitally recorded and transcribed verbatim by the first author (ADH).

### Data Analysis

Qualitative content analysis was conducted using a deductive approach in order to apply the five key concepts to the data as outlined by the Swedish Knowledge Governance Framework (i.e., patient safety, time-saving work methods, quality of care, equitable care and professional autonomy) to the data, followed by an inductive analysis in order to explore the concepts that were of significance to the participants. Deductive content analysis can be used when the researcher wishes to retest existing theories, models or concepts in a new context (Kyngäs et al., 2020), which suited the purposes of the present study since it is based on earlier theories or models and functions as an aid in moving from the general to the specific (Graneheim et al., 2017). The first step in the analytic process was to read the interview transcripts in full several times to become familiar with the data. All three authors read portions of the transcriptions in order to gain an overall impression and sense of the text. These close readings were underpinned by our understanding of the value of the hermeneutic circle in understanding the parts and the whole of a text to grasp its meaning and the importance of continually questioning interpretations throughout the analysis process (Gadamer, 2004). The next step was to construct a structured categorization matrix (Table 2) as proposed by Elo and Kyngäs (2008) so that the data could be framed according to the five key concepts. The transcripts were subsequently reviewed for content and meaning units in the participants’ statements, and preliminary codes were assigned to associated meaning units (Graneheim et al., 2017). The next step in the process was to integrate the codes into more comprehensive classes for subsequent categorization into sub-categories. This enabled aspects that do not fit the categorization framework to be used to create their own concepts, based on the principles of inductive content analysis (Elo & Kyngäs, 2008). These sub-categories were incorporated into the main categories of the matrix (i.e., the key concepts), however this inductive approach added contextual findings to our data that were not entirely obvious in the first deductive phase. The codes and sub-categories were considered on and discussed throughout the process using the categorization matrix. The first author (ADH) took the lead in the process of coding and categorizing, while the other authors (KM and TH) contributed to the process at discussion meetings by acting as co-analyzers of the text transferred into the categories in the matrix.

**Table 2.** Example of Categorization Matrix.

Category	Subcategory	Code	Meaning unit
Patient safety	The documentation template as a support	The documentation template to support the meeting with the patient	*. . .I have the template in front of me when I am with the patient. I look at it in detail, and if I get stuck on what to ask, I can take a look at it.*. . .*Then I*’*ve got all the keywords to hand. I have it next to me.*
Time-saving work procedures	Working as a new nurse using the template	Need for support as a new nurse	*. . .when I was very new, I asked all COPD patients about their allergy anamnesis, but exposure at work was more important. You learn that over time, you don*’*t let the the documentation template guide the contact with the patient as much.*
Equitable care	Conditions for follow-up	You ask the same questions based on where you live but you may not be able to follow up in the same way.	*. . .whether you receive the same care? Well, if you go through the documentation template, you have to ask the same questions and then you also obtain information about how the patient is doing and if they have the right compliance and so on.*

### Ethical Considerations

All nurses gave their written consent to participate and were informed that they had the right to withdraw at any time. The participants were assured confidentiality as the interview material was securely stored, with the first author having sole access to it. All participants were assigned a code to ensure their anonymity throughout the process, which was taken into account when reporting the findings. As there was no risk of physical or psychological harm, and no sensitive data was processed pertaining to individuals was, this study was not subject to ethical review in accordance with the Swedish Ethical Review Act (SFS 2003:460).

## Results

The findings indicate that the participating nurses experienced structured and standardized documentation to have both benefits and barriers. The content analysis identified, 11 sub-categories, as summarized in Table 3.

**Table 3.** Overview of Categories and Sub-Categories.

Patient safety	Time-saving work procedures	Quality of care	Equitable care	Professional autonomy
The documentation template as a support	Time-saving approaches	Standardized approaches	Follow-up conditions	Education and clinical experience
Objectives for documentation	Need for support as a new nurse	Aspects of fixed choices		Aspects of governance
Access to the documentation		A need for free-text documentation		

In this section, the findings are presented under each key concept with quotes underpinning the participants’ experiences: (1) patient safety; (2) time-saving work procedures; (3) quality of care; (4) equitable care, and (5) professional autonomy. All quotations have been translated from Swedish to English.

### Patient Safety

Patient safety has a central role in the documentation as it is a prerequisite for other care providers but additionally, the patient is able to read and understand information in the medical record. It must also be able to be possible to send the information to other systems and retain the meaning (Leotsakos et al., 2014).

With respect these goals of structured documentation most of the participants felt that the COPD documentation template served as a support and a memory aid during the visits and that the keywords and ready-made response options helped them to be confident that they included information relevant to the patient’s care.

*I always open the template before I bring the patient in and then I check that I have included most of the information. I think the template supports patient safety better than just a free text template* [Participant 4].

In the interviews, several participants reported a lack of knowledge regarding the purpose of certain keywords. Even though patient identity was a separate keyword in the template, few participants reported asking patients to identify themselves, only asking for first names in the waiting room. The majority of participants relied on the patient having registered themselves at the reception desk or through self-registration when documenting patient identity.

*Who wants to trick themselves into a spirometry examination? You usually just call out the first name due to confidentiality and it has also been the case here that the wrong patient has been admitted to the room, but you usually notice it very quickly.* [Participant 5]

Several participants felt that the COPD template was very thorough and that doctors and other healthcare professionals do not always have time to go through everything. However, it was noted that many patients accessed their documentation. The perception was that their patients seemed to understand the content of the asthma and COPD record but they sometimes received feedback and questions regarding records from other healthcare providers’

### Time-Saving Work Procedures

Structured data that can be easily transferred to other systems reduces information duplication and replaces manual entries, and it is expected, therefore, to be time saving (Ebbers et al., 2022). However, in this study, there was a lack of consensus about whether standardized documentation was time-saving. The general view was that documentation in relation to visits for asthma and COPD patients were time consuming. Despite this, most participants stated that the template saved time compared to documentation in a free text template.

*Yes, I think I save time compared to filling everything in in free text for this particular allergy test that was performed, which means that you don*’*t need to write it as free text, rather it is predefined and all you have to do is click to see what they are allergic to, so it*’*s great. And it is clear for doctors who might read it, as well as other healthcare professionals.* [Participant 4]

Some participants stated that, while it was helpful when they were new, it was time-consuming, to go through every single keyword in the template, as they did not have the experience to know what was relevant to the patient. Some also felt that it was difficult to write while having the conversation, which led them take handwritten notes and complete the EHR afterward. Nevertheless, almost all participants stated that the record template could help them during a stressful working day.

*In both stressful situations or if you*’*re new, it can be difficult to know what to ask about. “I forgot to check the oxygen saturation” or “oh, I didn*’*t ask about smoking” or whatever. You get support for how you should think, I reckon that’s better than being completely blank and coming up with everything yourself.* [Participant 1]

### Quality of Care

There is evidence that the quality of care can be improved through standardized and structured documentation that is based on evidence-based knowledge for certain patient groups (Ebbers et al., 2022). Participants in this study supported this, with some exceptions. Most participating nurses stated that the asthma and COPD template’s predetermined keywords help them work in a standardized way according to the guidelines for the patient group. However, several emphasized the importance of keeping the conversation flowing, despite following the template.

*I think it addresses most things and then some, but you have to read body language, listen and have follow-up questions. You can*’*t just ask yes and no questions like that, you also have to read between the lines, but it*’*s also an art.* [Participant 9]

A majority stated that aspects of fixed choices, predetermined keywords and checklist-based documentation enhanced quality. However, a couple of participants described negative aspects of using fixed yes/no answers and that in some cases these could be perceived as directly inappropriate.

*Maybe in the case of mental illness, spontaneously I wouldn*’*t feel that I would want a checklist because in those cases there is much more listening and asking follow-up questions depending on what the patient says. Do you have suicidal thoughts yes or no? You can*’*t do that. That would be too hard. Crises should not be a checklist, nor should dementia. It*’*s about psychological aspects. We are nurses, not machines.* [Participant 9]

*Fixed choices limit me, it gets a bit sloppy. We have a similar template in the triage context, it*’*s very black and white and that*’*s not what reality is like.* [Participant 5]

All participants felt that it was sometimes necessary to supplement the fixed choices with free text in the records, both to make the documentation more comprehensive and to avoid creating ambiguous notes.

*The patient says that they have had a difficult spring, had problems, felt isolated. It becomes a bit impersonal otherwise.* [Participant 2]

### Equitable Care

The use of structured documentation and standardized templates has been demonstrated to facilitate adherence to clinical guidelines, which can lead to more care equality (Andersson et al., 2023) and can be a means of reducing health disparities (Babyar, 2018). Benefits of care equality is something that was addressed by the participants of the present study.

*If one receives the same care, and if one goes through the checklist, one is obliged to ask questions and thereby also receives information about the patient*’*s condition, whether they have the correct technique, compliance, and so on. This provides more basis for making decision* [Participant 8]

Access to care metrics, such as nationally structured information for a particular patient group, allows for opportunities to improve quality on various levels and can provide data with a focus on groups at risk of being marginalized in their care (Yi et al., 2023) To target barriers to equitable health outcomes and improve equity, data quality is crucial for quality improvement efforts (Lewis et al., 2023).

In this study, a majority of the nurses stated that they and their organization did not have the time or opportunity to follow up on the SNAR’s statistics due to a pressurized work situation. They described it as unfortunate to not be able to work with register data for training or improvement work, especially since they spent a lot of time delivering the information due to long reception visits and time-consuming documentation.

*There is a lack of time available for the Swedish National Airway Register, a lack of time to access data for yourself, because it could do so much but we have not had the time at my job due to illness, my area is not a priority when colleagues are off sick.*[Participant 1]

### Professional Autonomy

Some standardized programs for patients can be perceived as challenges to professions, because professionals cannot apply their clinical view in the same way and make decisions according to patients’ individual differences (Cortés-Puch et al., 2020). Most of the participants appreciated having fixed choices and predetermined keywords, but very clearly emphasized the fact that training and experience are necessary to be able to make a decision about which parts of the documentation template are relevant to the individual patient. Most of the interviewees did not think that the record template controlled them once they had developed their professionalism, by which time they had the security of being able to skip things. They also emphasized the importance of being able to ask follow-up questions outside the template.

*You have to use your brain even though you have a template, you can*’*t just look at the template because there are other follow-up questions that are not included in it.* [Participant 3]

A few nurses discussed the risks of having too much standardization as it can sometimes be difficult to think more broadly.

*A bit like losing your autonomy, it*’*s both positive to have a system that can be followed straight away and that is the same for everyone, but at the same time you might lose some of this sensitivity when you have to ask all patients the same questions.* [Participant 2]

Asthma and COPD nurses generally work very independently as they have specialist nursing clinics and carry out their own annual checks. They often have contact with other professions such as doctors, dieticians and physiotherapists, but they are usually the ones who have the best insight into national guidelines. Several participants noted that a documentation template based on guidelines can support nurses in providing good care to patients in collaboration with other professional categories.

*Since there are clear guidelines, I can also tell the doctor how I have arrived at my position, assessment, decision and recommendation. Whether it should be changed and so on. I*’*m fairly new to COPD and asthma, so there are still a lot of pieces of the puzzle missing, but I notice that many people in the diabetes section have very antiquated knowledge and ways of working. Support in the documentation or in guidelines means that I can tell the doctor that this is how it is and therefore you must do this. So it is much clearer.* [Participant 8]

## Discussion

The objective of this study was to examine primary care nurses’ experiences with structured EHR documentation and its direct transfer for quality purposes within their daily work. Based on key concepts pivotal for structured and standardized documentation, as outlined by the Swedish Knowledge Governance framework, the results underline the fact that direct transfer of structured documentation for quality purposes in primary care management of COPD patients has the potential to enhance equitable care and safety. Professional experience and autonomy were described by the nurses as important prerequisites to achieve these benefits.

Several studies indicate that the time nurses spend on clinical documentation has increased significantly (Bøgeskov & Grimshaw-Aagaard, 2019; Cooper et al., 2021), reducing time for dedicated patient care. Nevertheless, the nurses in this study were ambivalent about whether structured documentation is time-consuming or time-saving. Less experienced nurses felt that templates led to lengthy and comprehensive clinical interviews, suggesting a need for new nurses to receive training in understanding the templates’ purposes and using them effectively. Such training might also encourage new nurses to move beyond the template when needed, as described by more experienced nurses.

Structured documentation can reduce documentation time and improve workflows (Ebbers et al., 2022). However, there is some evidence that electronic documentation whilst talking to the patient might restrict eye-contact and have an adverse impact on the clinician-patient relationship (Alkureishi et al., 2016; Rathert et al., 2017), compared with paper-based notes. Although structured documentation results in lengthier notes (Ebbers et al., 2022), several reports demonstrate improvements in documentation quality and organization (Cooper et al., 2021; Ebbers et al., 2022; Khan et al., 2022). This implies that the quality of data automatically extracted to quality registries might also improve, which is a crucial component for enhancing health care and identifying barriers to health equity. However, the participants did not perceived this process to be time-saving. The stressful primary care environment may explain why nurses rarely used COPD registry data for learning and quality improvement, despite the templates being designed for this purpose. It is noteworthy that the registry resources are underused by all participating clinics. From the primary care nurses’ perspectives, the benefits of automated reuse of EHR healthcare data are not apparent if they are not utilized.

Nurses described the templates as aids that reduced the risk of forgetting clinically relevant questions during COPD patient interviews, a benefit supported by recent studies (Ebbers et al., 2022). It is debatable whether these procedures reduce the risk of excluding certain questions based on caregivers’ own norms, values, or unconscious prejudices. Further, as shown by Vest et al. (2017), the inclusion of unstructured data is important in identifying patients with complex needs who may benefit from services that address social determinants of health that significantly influence health outcomes.

Professional autonomy and experience were important factors affecting the participants’ perceptions of quality in their clinical encounters with COPD patients. While the templates provided valuable guidance, they could be limiting, requiring nurses to ask follow-up questions and include free text data. Certain information, such as comorbidities and complex issues, may be better suited for unstructured documentation (Jefferies et al., 2010). More research is needed on the quality of structured information extracted from free text notes (Liu & Edye, 2020; Sezgin et al., 2023).

Relating to patient safety, several nurses noted that many patients read their personal COPD medical records. Patients sometimes had queries and asked questions about documentation from other clinicians, indicating that EHR enhances patient-provider interaction and participation, key to patient-centered care (Bøgeskov & Grimshaw-Aagaard, 2019).

Despite the importance of clinical documentation, there are indications that a great deal of time is being spent on documentation (Bøgeskov & Grimshaw-Aagaard, 2019). With studies indicating that it can take up more than 40% of nurses’ time on documentation (De Groot et al., 2022; Schenk et al., 2017). The rising demand for healthcare data extraction from both structured and unstructured documentation may increase documentation workloads and dissatisfaction among staff. The study highlights the need for effective introduction and understanding of templates in primary care, potentially through educational programs. The results also highlight the ongoing discussion concerning the fact that healthcare managers and stakeholders need to evaluate whether there is a threshold for the amount of healthcare data that provides value relative to the administrative burden it creates

### Methodological Considerations

This study has some limitations, which we report here. Our sample was subject to some general limitations of self-selection, for example, that those who volunteered to participate might have been more likely to be interested and invest in the topic. Only one man was included in the study, which is, however, consistent with the gender balance among COPD nurses in Sweden. Interview data were collected from COPD nurses working in Swedish primary care, and some caution regarding the transferability of our findings must thus be considered in relation to organizational and cultural differences. In addition, this study explores the perceptions of healthcare nurses without any further inquiry into patients’ perspectives.

The choice to use a hermeneutic method for the study can be questioned, as a phenomenological method might have been more suitable. The decision to adopt the hermeneutic perspective was influenced by one of the authors’ pre-understanding as an asthma and COPD nurse, consisting with the notion that a person seeking to understand a subject matter already has a bond to it. Recognizing this potential influence on the analysis was therefore considered more transparent.

The study also has some significant strengths. First, the study’s sampling derived from four diverse county councils in Sweden, thus reinforcing the credibility of the results. Second, the interviews and preliminary analyses were conducted by a single researcher, ensuring the consistency of the data collected. However, all three authors contributed to the data analysis, which enhanced study dependability. Furthermore, to improve dependability and confirmability, a detailed description of the research procedure was provided, enabling similar studies in the future. The reflections on the connections between the researcher, who is a COPD nurse, and the context during both data collection and analysis enhanced the trustworthiness of the study. This alignment ensured a deeper understanding of the data and maintained the integrity of the findings, as described by Elo and Kyngäs (2008).

## Conclusion

The nurses in this study experienced some barriers to structured documentation, but they also discovered numerous opportunities linked to providing comprehensive and safe care for patients with COPD in primary care. In achieving these benefits, professional experience and autonomy were considered important prerequisites, as there was a need to look beyond the template in the clinical conversations and to occasionally add free text information. Nurses stated that a thorough introduction was needed to the structured template for documentation in order to increase work efficacy, although documenting during the patient interview was an absolute must, which in some cases might affect clinician-patient relationships. By incorporating study findings into practice, work procedures and introduction of structured documentation among nurses might be improved. The relevance of this study might be transferred to other healthcare contexts where structured documentation is applied and reused for quality purposes.

Improvement of data extracted from the clinical setting is promising, however increasing requirements to capture more data while providing care means less time to use data for learning and quality improvement. This raises further questions concerning clinicians’ perceptions regarding documentation workload in relation to both quality and usefulness of structured and unstructured data.

## Appendix

**Appendix 1.** Interview Guide.

*Please, tell me more about your clinical documentation workflow
*Please describe you general feelings about the COPD documentation template
*Can you tell me about how you experience structured documentation as compared to more general keywords and free text?
*Are you experiencing any challenges with the fixed keywords?
*If you were a new nurse, how would you experience the structured documentation?
*Can you describe any barriers or facilitators in your meetings with patients that are associated with structured documentation?
*Please describe your present and previous documentation procedures in relation to quality of care.
*Please describe your present and previous documentation procedures in relation to patient safety?
*Do you miss something in asthma and COPD documentation templates?
*Please describe if you have access or work with data from the Swedish National Airway Register? Is it used by your clinic?
*Can you tell me about the time spent on clinical documentation? In relation to structured/unstructured documentation.

## Statements and Declarations

**Data Availability:** The data that support the findings of this study are available on request from the corresponding author.

**Guarantor:** KM
